# Photoreduction of CO_2_ to CH_4_ over Efficient Z-Scheme *γ*-Fe_2_O_3_/g-C_3_N_4_ Composites

**DOI:** 10.1155/2022/1358437

**Published:** 2022-04-26

**Authors:** Thanh-Binh Nguyen, Thuy Hang Dinh Thi, Doan Pham Minh, Hien Bui Minh, Ngoc Quynh Nguyen Thi, Bang Nguyen Dinh

**Affiliations:** ^1^VNU University of Science, Hanoi, Vietnam; ^2^Vietnam Maritime University, 484 Lach Tray, Hai Phong, Vietnam; ^3^IMT Mines Albi, Campus Jarlard, Albi CT Cedex 09 81013, Albi, France; ^4^Vietri University of Industry, Tien Kien, PhuTho, Vietnam

## Abstract

A series of composite *γ*-Fe_2_O_3_/g-C_3_N_4_ (denoted as xFeCN with *x* equal 5, 10, 15, and 20 of *γ*-Fe_2_O_3_ percentage in weight) was prepared by calcination and precipitation-impregnation methods. X-ray diffraction (XRD), Fourier transform infrared (FTIR), and X-ray photoelectron spectrometry (XPS) characterizations indicated the successful synthesis of Z-scheme FeCN composites. A red shift of the light absorption region was revealed by UV-vis diffuse reflectance spectroscopy (UV-DRS). In addition, photoluminescence spectroscopy (PL) spectra showed an interface interaction of two phases Fe_2_O_3_ and g-C_3_N_4_ in the synthesized composites that improved the charge transfer capacity. The photocatalytic activity of these materials was studied in the photoreduction of CO_2_ with H_2_O as the reductant in the gaseous phase. The composites exhibited excellent photoactivity compared to undoped g-C_3_N_4_. The CH_4_ production rate over 10FeCN and 15FeCN composites (2.8 × 10^−2^ and 2.9 × 10^−2^ *μ*mol h^−1^ g^−1^, respectively) was *ca.* 7 times higher than that over pristine g-C_3_N_4_ (0.4 × 10^−2^ *μ*mol h^−1^ g^−1^). This outstanding photocatalytic property of these composites was explained by the light absorption expansion and the prevention of photogenerated electron-hole pairs recombination due to its Z-scheme structure.

## 1. Introduction

Carbon dioxide from fossil energy consumption is the most important source of greenhouse gas emissions to the atmosphere, causing global warming [1, 2]. Carbon dioxide capture and storage (CCS) or utilization (CCU) has largely been studied during the last decades [3, 4]. Among them, photocatalytic CO_2_ conversion into valuable compounds, such as CH_4_ and CH_3_OH, is an attractive route [5–7]. Thus, many semiconductors have been investigated as photocatalysts with a particular focus on the development of photocatalytic heterojunction systems by combining various semiconductor materials to form different photocatalyst types such as type I, type II, or especially Z-scheme systems [8–12]. In the type I and type II photocatalysts, there occurs the photogenerated electrons transfer between conduction bands and holes, one between valance bands of each component in a composite. While in the Z-scheme type, this transfer takes place between the conduction and valance band of two adjacent components. By this characteristic, the Z-scheme photocatalyst has the advantage of mobilizing the potential position of the conduction band or valance band of each semiconductor when they are simultaneously combined for targeted reaction. Thereby, Z-scheme photocatalysts are expected to have stronger redox properties, better charge transfer, and higher improved light absorption yield than those of other simple photocatalyst types.

Recently, graphitic carbon nitride, g-C_3_N_4_, a semiconductor, has attracted researchers as a potential photocatalyst thanks to its easy synthesis, low cost, and moderate bandgap energy of 2.7 eV, which allows the absorption of visible light [13, 14]. In particular, the conduction band (CB) position of g-C_3_N_4_ is quite negative (−1.14 eV), which is favorable for the photoreduction of CO_2_ into almost valuable hydrocarbons such as CH_4_, HCOOH, CH_3_OH, and C_2_H_5_OH [15]. However, one of the major disadvantages of g-C_3_N_4_ is the fast electron-hole pair recombination, the relative high bandgap energy to be able to absorb most of the visible light, occupying 44% solar spectra. To improve these drawbacks, one of the strategies is to combine g-C_3_N_4_ with other semiconductors [16–20].

Among the semiconductors used as photocatalysts, iron oxide, Fe_2_O_3_, is an interesting candidate [21]. One of the outstanding features of this semiconductor is its low synthesis cost and relatively low band gap energy, *E*_g_ = 2.2 eV, which allows broad absorption in the visible region of sunlight. Therefore, Fe_2_O_3_ oxide has been extensively studied as a photocatalyst through the photodegradation of polluted organic compounds in water [22, 23]. However, for the photoreduction of CO_2_, the conduction band potential (CB) is positive (+1.58 eV). So, Fe_2_O_3_ oxide is not able to reduce this molecule. Combining Fe_2_O_3_ oxide with another semiconductor having enough negative conduction band potential is required to create an efficient photocatalyst for CO_2_photoreduction [24–29]. The improving photocatalytic activity by adding *γ*-Fe_2_O_3_ was also reported in some other works. In the research of Ding et al., for the photoreduction of CO_2_ in liquid phase to CH_3_OH [30], *a*-Fe_2_O_3_/g-C_3_N_4_ with the weight ratio *a*-Fe_2_O_3_: g-C_3_N_4_ of 60 : 40 had the best photocatalytic activity with 2.9-fold than pristine g-C_3_N_4_. Duan and Mei [31] also reported the highest amount of CH_3_OH obtained on 15%Fe_2_O_3_/g-C_3_N_4_(>3.5 fold than pristine g-C_3_N_4_). For CO_2_ photoreduction in the gas phase, Wong et al. [32] observed the CO formation on dendrite-structured *a*-Fe_2_O_3_/g-C_3_N_4_ (27.2 *μ*mol h^−1^g^−1^), which was about 2.2 times higher than the one on pure g-C_3_N_4_ (10.3 *μ*mol h^−1^ g^−1^). On the same type of catalyst with urchin-like *a*-Fe_2_O_3_ morphology, Yong Zhou et al. also recognized a CO production rate of 17.8 *μ*mol g^−1^ h^−1^, 3 times higher than that of pristine g-C_3_N_4_ (6.1 *μ*mol g^−1^ h^−1^) [33]. Besides, Fe_2_O_3_/g-C_3_N_4_ composites also showed higher photoactivity than pure *a*-Fe_2_O_3_ and g-C_3_N_4_ in the photodegradation of organic pollutants (Direct Red 81, Rhodamin B, and tetracycline hydrochloride) [34–36]. These studies showed that the morphology of Fe_2_O_3_ oxide seems to play an important role in the activity of catalyst. The different morphologies may change interface interaction between Fe_2_O_3_ and g-C_3_N_4_ phase, leading to the change in charge carrier, a key factor of photocatalysis.

Based on the analysis above, in this study, we have synthesized Z-scheme *γ*-Fe_2_O_3_/g-C_3_N_4_ photocatalysts for CH_4_ production from CO_2_ photoreduction in the gas phase. This new Z-scheme materiel could take advantage of the low bandgap energy of *γ*-Fe_2_O_3_ oxide and the rather negative conduction band potential of g-C_3_N_4_ as well.

## 2. Experimental

### 2.1. Materials

Melamine (C_3_H_6_N_6_), iron (II) sulfate heptahydrate (FeSO_4_.7H_2_O), and sodium hydroxide (NaOH) with analytical purity were purchased from Sigma-Aldrich. Deionized water was used as solvent for all preparations.

### 2.2. Synthesis of g-C_3_N_4_ and *γ*-Fe_2_O_3_/g-C_3_N_4_ Composite

Carbon graphitic nitride, g-C_3_N_4_, was synthesized by calcination of melamine at 550^o^C for 3 hours under the nitrogen atmosphere.

A series of x%*γ*-Fe_2_O_3_/g-C_3_N_4_ composites was prepared by the precipitation-impregnation method. First, 1 g of g-C_3_N_4_, which were synthesized from melamine calcination, was added in a 100 ml solution of 0.1 M NaOH. The mixture was covered by paraffin paper, stirred, and kept at 60^o^C for 2 hours. Then, a calculated amount of FeSO_4_.7H_2_O was slowly added in the solution above, and the pH was adjusted to 10 with 0.1 M NaOH solution, leading to the formation of a composite material of iron oxide precipitate and g-C_3_N_4_. The latter was filtered and washed by deionized water and dried at 70^o^C. Four Z-scheme *γ*-Fe_2_O_3_/g-C_3_N_4_ photocatalysts, denoted as xFeCN, with *x* equal to 5, 10, 15, or 20 wt.% of *γ*-Fe_2_O_3_ were obtained.

### 2.3. Characterization

All composites were characterized by X-ray diffraction (XRD, model Bruker D8), Fourier transform infrared (FTIR, model 8101M Shimazu), X-ray photoelectron spectroscopy (XPS, Thermo VG Scientific MultiLab 2000), UV-vis diffuse reflectance spectroscopy (UV-DRS, model Jaco V-530), photoluminescence spectroscopy (PL, model Horiba FluoroMax-4), transmission electron microscopy (TEM, JEM 1400, Plus Jeon), and scanning electron microscopy-energy dispersive X-ray spectrometry (SEM-EDS, Hitachi TM4000 Tabletop Microscope).

### 2.4. Photocatalytic Procedure

**Figure 1:**
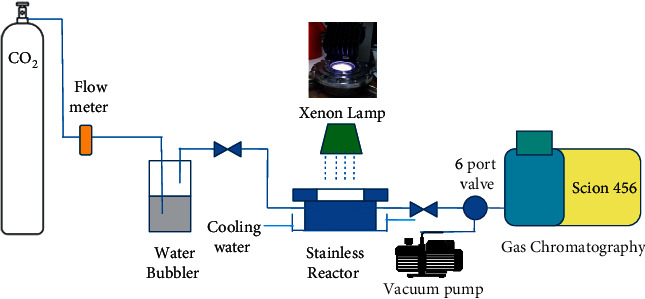
Scheme of the photocatalytic reactor used in this work.

In a typical test, 100 mg of catalyst was added in 15 mL deionized water in a beaker with a 5 cm diameter. The solvent in the mixture was completely evaporated in an oven at 70^o^C to well disperse the photocatalyst on the bottom of the beaker. After that, the catalyst-containing beaker was placed in a closed stainless reactor equipped with a quark window (Figure 1). The high purity (99,999%) CO_2_ flow of 500 ml/minute bubbled in a closed stainless contains deionized water kept at 25^o^C before entering the stainless reactor for 30 minutes. Then, a 150W Xenon lamp (Newport model 67005) was switched on for 18 h.

## 3. Results and Discussion

**Figure 2:**
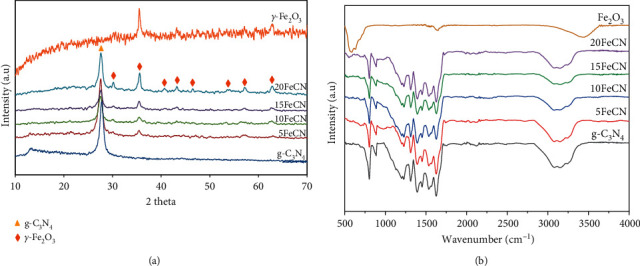
XRD patterns (a) and FTIR (b) spectra of g-C_3_N_4_, xFeCN composites.

Figure 2(a) shows the XRD patterns of the fresh g-C_3_N_4_ and xFeCN photocatalysts. The formation of g-C_3_N_4_ by melamine calcination was confirmed by the presence of peaks at 2*θ* of 13.2 and 27.3° (JCPDS 87–1526). For the composite photocatalysts, the characteristic peaks of *γ*-Fe_2_O_3_ appeared in all patterns with 2*θ* at 30.2, 35.7, 43.2, 53.8, 57.2, and 62.9° (JCPDS 004–075). The peak intensity of the *γ*-Fe_2_O_3_ phase is proportional to its content in the composite materials.

**Figure 3:**
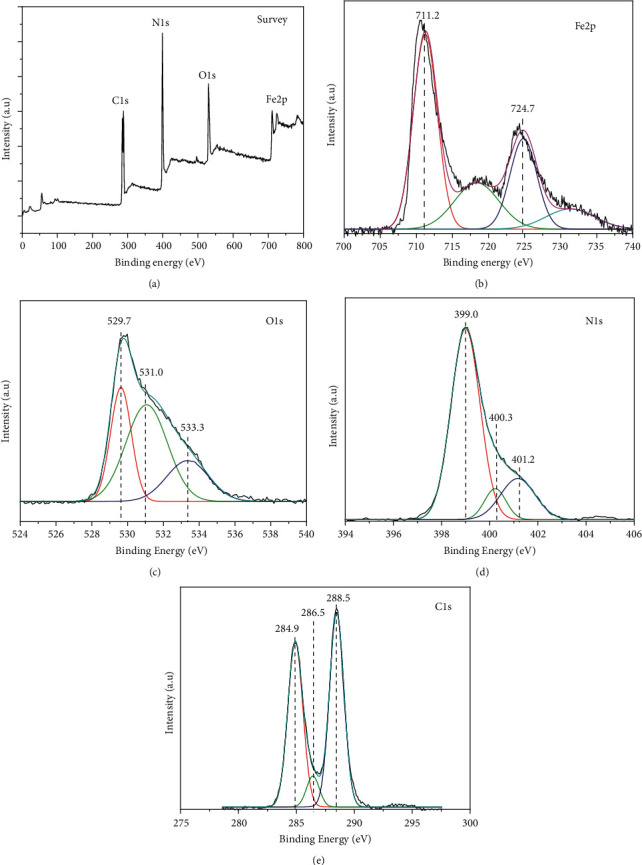
XPS survey of 10FeCN composite (a) and its XPS spectra of Fe2p (b), O1s (c), N1s (d), and C1s (e).

Figure 2(b) shows the FTIR spectra of all fresh photocatalysts. All these materials have similar characteristic signals of g-C_3_N_4_ structure. The peak at 804 and 887 cm^−1^ is attributed to the heptazine stretching mode [37], while those at 1203–1630 cm^−1^ correspond to stretching vibrations of C=N and C–N bonds of the heterocycle [38, 39]. For the broad band from 3000 to 3500 cm^−1^, the signals were ascribed to the vibration of NH_2_ and NH functional groups of g-C_3_N_4_ and the OH group of absorbed water [40]. In addition, the weak signals observed at586 cm^−1^ originated from the Fe–O bond vibrations [41]. These results confirmed the formation of the two expected crystalline phases of g-C_3_N_4_ and *γ*-Fe_2_O_3_ in the photocatalysts synthesized. In order to confirm the formation of composite materials between these two components, XPS analysis was performed for one photocatalyst containing 10 wt% of *γ*-Fe_2_O_3_ (Figure 3). The general XPS spectrum (Figure 3(a)) shows the presence of Fe, O, C, and N on the surface of this photocatalyst. On the high-resolution Fe elemental spectrum (Figure 3(b)), there are two major peaks at the binding energy 711.2 and 724.7 eV corresponding to Fe^3+^2*p*_3/ 2_ and Fe^3+^2*p*_1/2_, respectively. These two peaks are characteristics of the Fe^3+^ oxidation state as observed on the XPS spectrum of Fe_2_O_3_ oxide [42, 43]. Besides, two satellite peaks can be detected at positions 718.2 and 731.0 eV. With the C1s spectrum (Figure 3(e)), there are three peaks at 284.9, 286.5, and 288.5 eV. The first peak at 284.9 eV is assigned to C–C/C=C bonds, the second to the carbon of the C–NH_2_ group, and the third to bonded C in the heptazine structure (N–C=N) [44, 45]. In the N1s spectrum (Figure 3(d)), the peak deconvolution shows the presence of 3 peaks at 399.0, 400.3, and 401.2 eV, which correspond to Sp^2^ bonded N in triazine ring (C–N=*C*), the tertiary nitrogen N-(C)_3_ group in the heptazine structure, and N in the C–N–H group, respectively [39, 40]. For the oxygen element (Figure 3(c)), the peak deconvolution of O1s indicates the existence of three components: lattice oxygen of Fe–O bond (529.7 eV), oxygen of surface hydroxyl–OH (531.0 eV), and the one of adsorbed water H_2_O (533.3 eV) [43]. Hence, the results of XPS, XRD, and IR proved that *γ*-Fe_2_O_3_/g-C_3_N_4_ composites were successfully synthesized.

**Figure 4:**
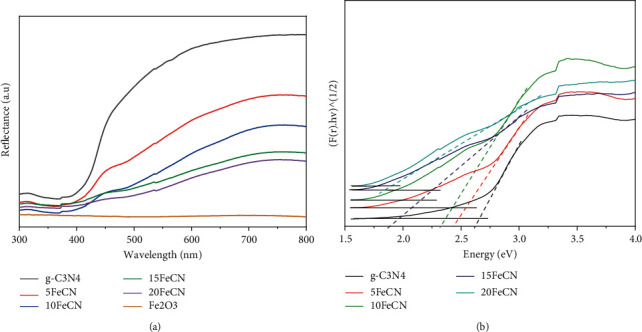
UV-DRS spectra (a) and Kubelka–Munk function (b) of xFeCN composites.

In order to predict the light absorption ability, all composites and g-C_3_N_4_ were measured by the UV-DRS method. Figures 4(a) and 4(b) show the obtained results. From the Kubelka–Munk function, the obtained bandgap energy was of 2.63, 2.54, 2.47, 2.25, 1.95, and 1.80 eV for g-C_3_N_4_, 5FeCN, 10FeCN, 15FeCN, 20FeCN, and *γ*-Fe_2_O_3_, respectively. So, the increase of *γ*-Fe_2_O_3_ content led to decrease of the bandgap energy. In other words, it means that the light absorption region of catalysts shifted more in the visible light wavelength by increasing the *γ*-Fe_2_O_3_ content. Hence, the photocatalytic activity of composites is expected to be improved.

**Figure 5:**
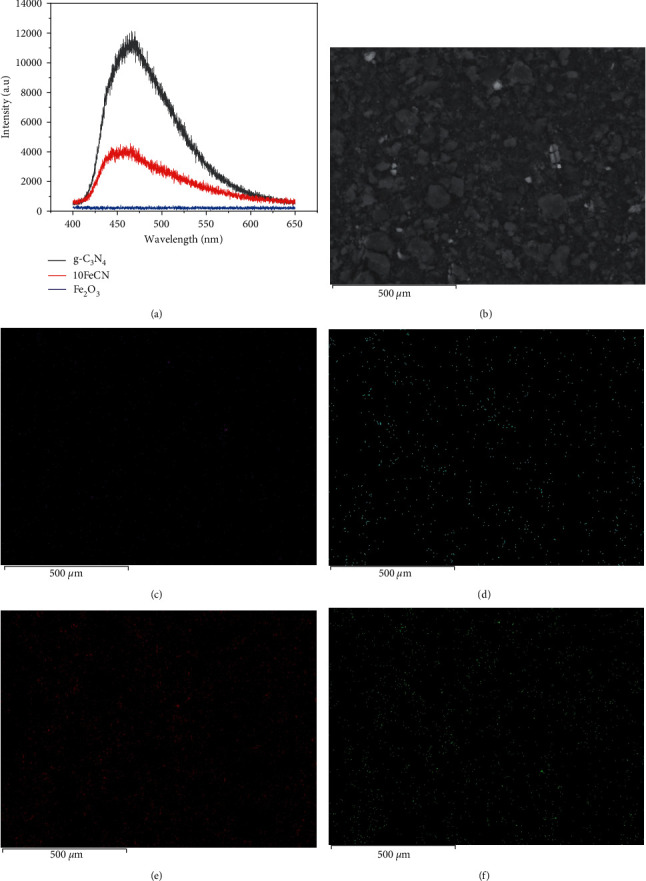
PL spectra of g-C_3_N_4_, *γ*-Fe_2_O_3_, and 10FeCN composite. (a) SEM mapping images of different elements in 10FeCN: SEM image (b), Fe distribution (c), O distribution (d), C distribution (e), and N distribution (f).

To estimate the recombination of electron-hole pair phenomena, the PL spectroscopy was carried out for g-C_3_N_4_, *γ*-Fe_2_O_3_, and 10FeCN composites (Figure 5(a)). As observed, the intensity of PL spectrum of g-C_3_N_4_ is nearly twice in comparison with the one of 10FeCN. No signal was observed on PL spectra of Fe_2_O_3_ at activated wavelength. This result reflected that the presence of *γ*-Fe_2_O_3_ and its interface interaction with g-C_3_N_4_ seem to inhibit the photogenerated electron-hole pairs recombination, which could improve photoactivity.

Figures 5(b), 5(c), and 5(d) show the SEM image and different element distribution of the 10FeCN sample. It is obvious that Fe and O elements or Fe_2_O_3_ phase were quite homogenously dispersed on the surface of g-C_3_N_4_. The elemental composition is given in Table 1. It is noted that the experimental composition is quite.

**Figure 6:**
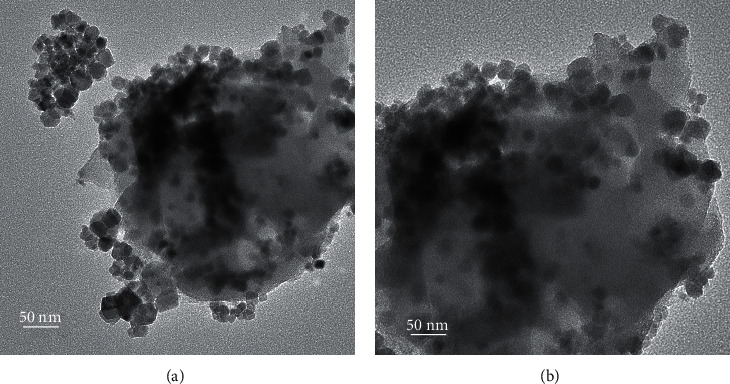
TEM images of 10FeCN.

The morphology of composite 10FeCN was characterized by TEM (Figure 6). It is obvious that the particle Fe_2_O_3_ is in cubic form with average size of 10 nm. Apart from *γ*-Fe_2_O_3_ particles dispersed on the g-C_3_N_4_ surface, it seems that some these particles were covered by g-C_3_N_4_ layer to form core-shell structure Fe_2_O_3_@g-C_3_N_4_.

**Figure 7:**
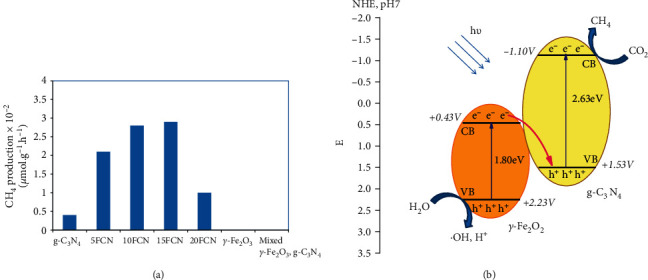
CH_4_ production on g-C_3_N_4_ and different xFeCN composites (a); proposed mechanism of CO_2_ photoreduction on Z-scheme structure of *γ*-Fe_2_O_3_/g-C_3_N_4_ (b).

The photocatalytic activity was evaluated through photoreduction of CO_2_.  Figure 7(a) shows the obtained results. Methane was the main production of the reaction, while CO was not noticeable. No product was detected on *γ*-Fe_2_O_3_. This is explained by the more positive value of its conduction band potential in comparison of that reduction potential of CO_2_/CH_4_ (−0.24 V) [2]. It was noted that the presence of *γ*-Fe_2_O_3_ remarkably impacted the CH_4_ formation. A volcano-like evolution of methane formation as a function of *γ*-Fe_2_O_3_ is observed. Concretely, under the same operational conditions, the amount of CH_4_ formed is 0.4 × 10^−2^, 2.1 × 10^−2^, 2.8 × 10^−2^, 2.9 × 10^−2^, and 1.0×10^−2 ^*μ*mol g^−1^ h^−1^ for g-C_3_N_4_, 5FeCN, 10FeCN, 15FeCN, and 20FeCN, respectively. Hence, the maximum CH_4_ formation quickly increased when rising the *γ*-Fe_2_O_3_ content and reached the maximum with 10FeCN and 15FeCN composites. Thus, compared to pristine g-C_3_N_4_, the produced amount of CH_4_ over 10FeCN and 15FeCN composites was 7 fold. However, at higher *γ*-Fe_2_O_3_ content, e.g., 20wt.% Fe_2_O_3_, CH_4_ production dropped rapidly to 1.0×10^−2 ^*μ*mol g^−1^ h^−1^. In comparison with the research on urchin-like Fe_2_O_3_/g-C_3_N_4_ and dendrite-structured Fe_2_O_3_/g-C_3_N_4_ catalysts for CO_2_ photoreduction in the gas phase, CH_4_ gas was the preferred product on this catalyst, instead of CO [32, 33]. Hence, the obtained results show interesting photocatalytic activity of the synthesized composite materials and also prove the good combination of two phases, *γ*-Fe_2_O_3_ and g-C_3_N_4_, to make new active photocatalysts. The small formed CH_4_ amount was probably due to the low power of xenon lamp (only 150W). To justify these results, a blank test (without catalyst) and a test on g-C_3_N_4_ under N_2_ gas were carried out. No CH_4_ amount was detected for these ones. In addition, the test was performed also on the mixture of 10% (wt) of *γ*-Fe_2_O_3_ with g-C_3_N_4_ (Figure 7(a)), and the CH_4_ product is under detectable limit that could be evidence for the absence of phase interaction between *γ*Fe_2_O_3_ and g-C_3_N_4_ as well as the presence of *γ*-Fe_2_O_3_ hindering the illumination on g-C_3_N_4_.

Generally, the outstanding photoactivity of *γ*-Fe_2_O_3_/g-C_3_N_4_ composite materials compared to g-C_3_N_4_ is assigned to better light-harvesting ability and charge transfer than those of single phase of *γ*-Fe_2_O_3_ or g-C_3_N_4_. It should be emphasized that the charge transfer process in the Z-scheme structure not only reduces photoelectron-hole pair recombination but also makes photocatalyst, the redox potential, stronger: CB potential more negative and VB potential more positive. In addition, it seems that the quite small size particles of *γ*-Fe_2_O_3_ as observed in TEM images, about 10 nm of diameter, have improved the interface interaction of composite *γ*-Fe_2_O_3_/g-C_3_N_4_. The increase of *γ*-Fe_2_O_3_ quantity superior of 15% (wt) has led to the decrease of photoactivity. This is probably due to the strong sintering of *γ*-Fe_2_O_3_ nanoparticles, causing less interface interaction. Besides, the accumulation of *γ*-Fe_2_O_3_ nanoparticles could cover g-C_3_N_4_ surface and inhibits the irradiation on this phase. Hence, the *γ*-Fe_2_O_3_ quantity of 10% (wt) was the most suitable to obtain the highest photoactivity.

From UV-DRS spectrum and Mulliken electronegativity theory, the valance band (VB) and conduction band (CB) of *γ*-Fe_2_O_3_ and g-C_3_N_4_ were calculated [30]. According to this theory, *E*_CB_ = *X*−*E*_c_−0.5*E*_g_, where E_CB_ is the conduction band edge energy, *X* is the electronegativity of the semiconductor (equal 5.825 eV for Fe_2_O_3_ and 4.72 eV for g-C_3_N_4_), *E*_c_, equal 4.5 eV, is the energy of free electrons with hydrogen scale, and *E*_g_ is the bandgap energy of semiconductor. Basing on this equation and concrete value of each parameter, it is found that VB and CB of *γ*-Fe_2_O_3_ and g-C_3_N_4_ are +2.23, +0.43 eV and +1.53, −1.10 eV, respectively.  Figure 7(b) shows a possible photoreduction mechanism of CO_2_ photoreduction into CH_4_. According to this Z-scheme composite, all the two phases, *γ*-Fe_2_O_3_ and g-C_3_N_4,_ harvested the irradiated light and generated electron-hole pairs. The photogenerated electron located on CB of *γ*-Fe_2_O_3_ migrated and recombined with photogenerated holes located on the valance band (VB) of g-C_3_N_4_. This process allows broadening the light absorption region and also prevents recombination of photogenerated electron-hole pairs that both improved the photocatalytic activity. The details of this process are presented through the following equations:

(1) $$\begin{array}{c} \gamma -{\text{Fe}}_{2}{\text{O}}_{3}/\text{g}-{\text{C}}_{3}{\text{N}}_{4}+\text{h}\nu ⟶\gamma -{\text{Fe}}_{2}{\text{O}}_{3}\left(h_{\text{VB}}^{+}\right)+\text{g}-{\text{C}}_{3}{\text{N}}_{4}\left({\text{e}}_{\text{CB}}^{-}\right), \end{array}$$

(2) $$\begin{array}{c} {\text{CO}}_{2}+8{\text{H}}^{+}+8{\text{e}}_{\text{CB}}^{-}\cdot \left(\text{g}-{\text{C}}_{3}{\text{N}}_{4}\right)⟶{\text{CH}}_{4}+2{\text{H}}_{2}\text{O}, \end{array}$$

(3) $$\begin{array}{c} 8{\text{H}}_{2}\text{O}+8{\text{H}}_{\text{VB}}^{+}\left(\gamma -{\text{Fe}}_{2}{\text{O}}_{3}\right)⟶8{\text{H}}^{+}\cdot +8\cdot \text{OH,} \end{array}$$

(4) $$\begin{array}{c} 8\cdot \text{OH}⟶4{\text{H}}_{2}\text{O}+2{\text{O}}_{2}. \end{array}$$

## 4. Conclusions

In this work, different Z-scheme photocatalysts, *γ*-Fe_2_O_3_/g-C_3_N_4,_ were synthesized by simple methods of calcination and impregnation-precipitation. XRD, IR, and XPS characterizations of these materials confirmed the formation of composite structure of these photocatalysts, wherein *γ*-Fe_2_O_3_ grew on g-C_3_N_4_ surface. SEM analysis highlighted a good distribution of Fe on the surface of g-C_3_N_4_ support. The UV-DRS and PL spectra of the photocatalyst containing 10 wt.% *γ*-Fe_2_O_3_ evidenced interface interaction of the two phases of *γ*-Fe_2_O_3_ and g-C_3_N_4_.

The catalytic performance of these materials was evaluated through the CO_2_ photoreduction in the gaseous phase. Methane was found as the main product, while no trace of CO was observed. According to CH_4_ production, the photocatalytic activity followed the following order: 15FCN (2.9×10^−2^ *μ*mol g^−1^ h^−1^)   10FeCN (2.8×10^−2^ *μ*mol g^−1^ h^−1^) > 5FeCN (2.1×10^−2^ *μ*mol g^−1^ h^−1^) > 20FeCN (1.0 × 10^−2^ *μ*mol g^−1^ h^−1^) > g-C_3_N_4_ (0.4×10^−2^ *μ*mol g^−1^ h^−1^). Hence, 10FeCN and 15FeCN composites had the highest photoactivity, which is approximately 7 times higher than that on bulk g-C_3_N_4_. This could be explained by a synergy combination and interaction of the two phases *γ*-Fe_2_O_3_ and g-C_3_N_4_, leading to the Z-scheme structure composites, which improved light absorption with red-shift light and also diminishes photogenerated electron-hole pairs recombination. These outstanding results are promising for the design of a low-cost and highly efficient photocatalyst for CO_2_ reduction on the basis of *γ*-Fe_2_O_3_ and g-C_3_N_4_.

## Figures and Tables

**Table 1:** Weight percentages of different elements in the composition of 10FeCN.

Element	Theoretical percent	Experimental percent
Fe	6.99	7.26
O	3.01	3.24
C	35.22	36.57
N	54.78	52.93

## Data Availability

The data used to support the findings of this study are included within the article.
